# Predictive Factors for a Long Postoperative Stay after Emergency Laparoscopic Cholecystectomy Using the 2013 Tokyo Guidelines: A Retrospective Study

**DOI:** 10.1155/2019/3942584

**Published:** 2019-04-16

**Authors:** Koichi Inukai

**Affiliations:** Department of Surgery, Kariya Toyota General Hospital, 5-15 Sumiyoshi-cho, Kariya, Aichi 448-8505, Japan

## Abstract

Laparoscopic cholecystectomy (LC) is widely used for treating early acute cholecystitis (AC) and substantially reduces hospital costs. This study aimed to identify and evaluate risk factors associated with long postoperative hospital stays (PHSs) in patients undergoing emergency LC for AC according to the 2013 Tokyo Guidelines (TG13). Clinical data of patients who underwent emergency LC for AC between 2011 and 2017 were retrospectively collected. Patients were divided into early discharge (ED, discharge in three days or less postoperatively) and late discharge (LD, discharge in more than three days postoperatively) groups based on clinical progression and PHS after LC. Preoperative characteristics and perioperative outcomes were analysed as potential risk factors for LD. Among 149 patients, 104 (69.8%) were discharged within 3 days postoperatively, whereas 45 (30.2%) had long PHSs. Main causes of LD were fever and inflammation. Univariate analysis of preoperative risk factors revealed significant differences in age, white blood cell count, C-reactive protein, total bilirubin (T-bil), and alkaline phosphatase (ALP) levels; anticoagulation therapy; and TG13 severity grade. Multivariate analysis revealed that TG13 severity grade II, age >65 years, and elevated T-bil and ALP levels are independent factors for long PHS. Older age, worse biliary function, and increased TG13 severity grade might predict prolonged PHSs in AC patients undergoing emergency LC.

## 1. Introduction

The safety and efficacy of laparoscopic cholecystectomy (LC), which has been increasingly adopted as a standard treatment approach [1], have been confirmed by numerous studies [2–4]. Furthermore, LC is recognised as a cost-effective treatment for patients with acute cholecystitis (AC) [4–6]. Zacks et al. [5] reported that LC significantly reduced hospital costs as well as length of stay and mortality compared with open procedures. Therefore, LC significantly improves the healthcare quality for patients with AC [5, 6].

Information is limited on specific factors affecting the length of postoperative hospital stays (PHS) associated with cholecystectomy for AC. Also, no studies have analysed long PHSs in patients undergoing emergency LC for AC according to the 2013 Tokyo Guidelines (TG13) [7].

Although several studies evaluated the associations among clinical factors including age, operation time, perioperative transfusion, and long PHS for elective LC [8, 9], no reports to date assessed the association between emergency LC and the length of PHS in patients with AC.

Recent studies revealed that total bilirubin (T-bil) and other liver function tests including aspartate aminotransferase (AST), alanine transaminase (ALT), and alkaline phosphatase (ALP) were predictors of common bile duct (CBD) stones in AC [10–13]. We hypothesised that biliary function test (BFT), that is, T-bil and ALP, was a predictor for delayed discharge. In the current study, we examined whether elevation of BFT was associated with delayed discharge and evaluated other potential risk factors for a longer PHS in patients with AC treated by emergency LC.

## 2. Methods

This retrospective clinical study was conducted at the Department of Surgery. This study was carried out in accordance with established national and institutional ethical guidelines regarding the involvement of human subjects and the use of human tissues for research. After obtaining approval from the IRB, the surgical records of all patients who underwent successful emergency LC between 2011 and 2017 at the study hospital were examined retrospectively. Emergency surgery was defined as less than 24 hours from admission to surgery.

According to the TG13 criteria [7], we performed emergency LC for patients with (1) American Society of Anesthesiologists (ASA) classification I or II; (2) within 72 hours after symptomatic onset; and (3) TG13 severity grade I or II [14]. All patients meeting this definition in the study period were included in the study analyses.

Patients with conversion from LC to laparotomy during surgery were excluded. Additionally, patients who underwent surgery for malignancy or with additional procedures planned at the time of LC were excluded. At the study institution, all patients with emergency LC were enrolled in a clinical pathway after surgery. In our clinical pathway for patients who underwent emergency LC, they were treated with antibiotics for the postoperative two days and planned to be discharged on postoperative day three.

Patients who were discharged in three days or less postoperatively (ED group) were compared with those who were discharged in more than three days postoperatively (LD group) to analyse factors predicting extension of PHS. To compare the characteristics of the groups, the following preoperative variables were extracted from the medical records: age, sex, body mass index (BMI), diabetes mellitus (DM), systemic anticoagulation therapy, history of laparotomy, white blood cell (WBC) count, C-reactive protein (CRP) level, haemoglobin, platelet count, BFT (normal ranges: T-bil ≤ 1.2 mg/dl, ALP ≤ 360 U/l), TG13 severity grade of AC, and time from onset to surgery. Also, the following variables were extracted: amount of blood loss, operation time, amount of crystalloid transfusion, and surgical complications (>grade II by the Clavien-Dindo classification [15]). Results were expressed as medians (range) unless otherwise stated. LD patients for only social reasons or patient preference was included in the ED group.

### 2.1. Laparoscopic Cholecystectomy

The surgical procedure was performed using a standard 30° laparoscope and four operating ports (a 10 mm umbilical port and three 5 mm ports). The three 5 mm ports were inserted in the right subcostal area. 5 mm laparoscopic coagulation shears were used for Calot's triangle dissection and cystic artery and duct isolation. Cystic artery control and cystic duct section after isolation were achieved using 5 mm laparoscopic clips. After cholecystectomy, 3-0 absorbable threads were used for closure of the fascia to prevent umbilical hernia.

### 2.2. Statistical Analysis

SPSS for Windows version 24.0 (SPSS, Chicago, IL, USA) was used for statistical analysis. The Mann–Whitney* U* test was used to compare continuous data between the two groups, whereas the chi-square or Fisher's exact test was used to compare categorical data. Logistic backward regression was performed to determine factors independently associated with LD. A p value < 0.05 was considered to indicate statistical significance.

## 3. Results

A total of 158 patients with grade I or II AC underwent emergency LC during the study period. Six patients whose surgery was converted to laparotomy intraoperatively, 2 patients with a possible malignancy diagnosis, and 1 patient who underwent LC with appendectomy were excluded. Therefore, the final analysis included 149 patients with a mean age of 36.8 years. There were 45 and 104 patients in the LD and ED groups, respectively. The median PHSs were 3 and 5 days in the ED and LD groups, respectively. The reasons for long PHSs included fever or inflammatory reaction (n = 18), monitor liver function (n = 12), drain removal (n = 4), postoperative ileus (n = 2), medical causes due to extreme old age (n = 8), biliary peritonitis due to perforated cholecystitis (n = 2), and postcholecystectomy syndrome (n = 1).

The patients' characteristics and operative outcomes are shown in Tables 1 and 2. The results of the univariate analysis for risk factors of LD are summarised. Briefly, demographic factors including age, WBC, CRP, BFT, systemic anticoagulation therapy, and TG13 severity grade II were significantly associated with LD. In contrast, sex, haemoglobin, platelet count, history of laparotomy, DM, and time from onset to surgery were not significant risk factors for LD.

The comparison of the operative outcomes showed that operation time, blood loss, postoperative complications, and transfusion of crystalloid fluid were significantly different between the groups. The postoperative complication rates were 8.89% (4/45) and 1.92% (2/104) in the LD and ED groups, respectively, which were not significantly different.

The multivariate analysis showed that TG 13 severity grade II (odds ratio [OR] 5.39; 95% confidence interval [CI] 2.26–12.90) compared to grade I, age over 65 years (OR 3.64; 95% CI 1.58–8.39), and elevated BFT (OR 2.69; 95%CI 1.17–6.20) were independent risk factors for a longer PHS (Table 3).

## 4. Discussion

AC is a common surgical diagnosis requiring patient admission. LC is the standard surgical intervention for patients with gallbladder disease and it is associated with lower postoperative pain because it is less invasive, allows earlier oral intake, enables an earlier return to normal activities, and shortens hospital stay [16–19].

The 2007 Tokyo Guidelines represented the first global classification system to diagnose and grade the severity of AC. Given the wide spectrum of gallbladder diseases, these guidelines are important for standardisation of scientific investigation and accurate prediction of clinical outcomes. These guidelines have been validated in Asian populations, and modifications have been made to improve the clarity of the diagnosis in the TG13 [19–22]. According to the TG13, early LC is the first-line treatment for grade I (mild) AC, whereas cholecystectomy may be indicated if advanced laparoscopic techniques are available for grade II (moderate) AC. Additionally, cholecystectomy is recommended to be performed soon after admission, particularly in patients admitted within 72 hours after the onset of symptoms. Therefore, at the study institution, patients with grade I or II AC underwent LC according to the TG13. Moreover, emergency LC was performed within 72 hours from the onset of symptoms and in only patients with ASA score I or II, because it is recommended that, in patients with surgical risk, observation (follow-up without cholecystectomy) after improvement with initial medical treatment could be indicated in guideline.

In a study by Murata et al. [23] who performed a large database analysis of 2176 patients with AC using Japanese big data, multiple linear regression analyses revealed that early and laparoscopic surgery was significantly associated with a decrease in the length of PHS, whereas preoperative antimicrobial therapy was significantly associated with an increase in the length of PHS. Hayasaki et al. [24] reported that performance status, WBC count, and TG severity grade were preoperative factors influencing PHS by multivariable analysis of the patients with AC. Paul et al. [25] analysed a retrospective cohort of patients with AC in the United States and found that independent predictive factors for increased length of PHS were TG13 severity grade, admitting service (medical or surgical), BMI, and WBC count. Although these studies did not distinguish between LC and laparotomy, preoperative factors influencing the length of PHS are TG13 severity grade, preoperative antimicrobial therapy, and WBC count.

WBC is a significant prognostic factor of AC [20, 26, 27], and the severity grade based on the TG13 includes WBC count as an index. Therefore, an increased WBC count and the TG13 severity grade are associated with prolonged PHS. Conversely, the current study revealed that, in addition to the TG13 severity grade, age over 65 years and elevated BFT were significant independent preoperative factors for a prolonged PHS. Although BMI is considered as one of the preoperative factors associated with conversion to open laparotomy for acute or chronic cholecystitis [28, 29], the current study excluded patients who were converted to laparotomy. Conversion to open laparotomy is a critical factor for long PHSs; therefore, the impact of BMI on the length of PHS in LC for AC might have been underestimated in this study.

Our working hypothesis that elevation of BFT was correlated with a prolonged PHS was demonstrated to be true in patients undergoing emergency LC for AC. Although CBD stones are not always present in patients with AC, AC is often associated with elevated liver function parameters for various reasons, including reactive hepatitis, portal tract inflammation, and occasionally direct pressure of the distended gallbladder on the biliary tract [30–33]. Recent studies reported that liver function tests including T-bil, AST, ALT, and ALP were predictors of CBD stones in acute cholecystitis [10, 11]. Although we assessed the absence of CBD stones by magnetic resonance cholangiopancreatography preoperatively in all cases, small CBD stones or biliary sludge, which may not be detectable by MRCP, might reside in the bile duct, with the possibility of obstructing the flow of purulent bile. As a result, inflammation might persist postoperatively, which was reflected by the presence of signs of inflammation in most patients in the LD group. A previous report suggested that antimicrobial prophylaxis may not be necessary for low-risk patients with AC undergoing LC [34]. Conversely, antimicrobial prophylaxis might be necessary to reduce PHS in only patients with AC and elevated BFT. Further clinical studies are warranted to develop guidelines for the preoperative use of antimicrobial drugs in these patients and to determine the early surgical approach that is suitable to minimise additional medical costs.

The current study has several limitations. A single hospital system may not accurately represent the general patient population or the laboratory findings. The findings of this retrospective study should be confirmed with well-designed prospective studies, including the elucidation of BFT as a predictor of a prolonged PHS in patients with AC. The analyses of the current study did not include AST, ALT, and glutamyl transpeptidase as they tend to be influenced by haemolysis and alcohol. The current study included all patients who underwent successful emergency LC surgery without open conversion. With broad inclusion criteria, a more accurate assessment of potential factors associated with a longer PHS is expected in further studies. With proper patient selection and adequate preoperative preparation, more patients might benefit from a shorter PHS.

## 5. Conclusions

Emergency LC is a useful and safe approach in AC; however, several factors might be associated with a longer PHS. The current study revealed TG13 severity grade and older age as strong independent risk factors for a prolonged PHS. However, given the association of preoperative BFT elevation with delayed discharge from the hospital, the significance of BFT in the length of PHS should not be ignored.

## Figures and Tables

**Table 1:** Patients' characteristics and univariate analysis for preoperative factors.

	ED group	LD group	P-value
	(*n* = 104)	(*n* = 45)	
Age (years)	54.5 (46.1–65.3)	65 (47.0–75.0)	0.015

Sex (%male)	71	32	0.879

BMI (kg/m^2^)	24.8 (22.3–26.8)	25.3 (23.5–27.6)	0.256

WBC (10^3^/*μ*L)	12.6(10.2–15.13)	15.2	0.007

CRP (mg/dL)	1.63 (0.36–6.62)	8.45 (1.87–16.17)	<0.001

HB (g/dl)	14.5 (13.5–15.3)	14.2 (12.7–15.4)	0.245

PLT (10^3^/*μ*L)	23.0 (20.1–27.6)	22.3 (17.9–26.1)	0.180

Past laparotomy, n	23	7	0.487

Elevation of BFT, n	25	22	0.005

Anticoagulation therapy, n	1	9	<0.001

DM, n	12	10	0.151

Grade I/II, n (%)	89(85.6)/15(14.4)	23(51.1)/22(48.9)	<0.001

Time from onset to surgery 0/1/2/3 day, n	23/46/28/7	6/24/11/4	0.548

BMI, body mass index; WBC, white blood cell; CRP, C-reactive protein; HB, haemoglobin; PLT, platelets; BFT, biliary function test; DM, diabetes mellitus.

Continuous variables are expressed as median (IQR).

**Table 2:** Univariate analysis for perioperative factors.

	ED group	LD group	P-value
	(*n* = 104)	(*n* = 45)	
Blood loss (ml)	10 (4–25)	50 (10–120)	<0.001

Operation time (min)	111 (92–140)	146 (128–174)	<0.001

Crystalloid transfusion (ml)	1500 (1200–1700)	2000 (1600–2400)	<0.001

Complication (n)	2	4	0.068

Continuous variables are expressed as median (IQR).

**Table 3:** Results of multivariate analysis for predictive factors.

Preoperative factor	Odds ratio	95% CI interval	P-value
Grade II	5.39	2.26–12.9	<0.001

Age >65 years	3.64	1.58–8.39	0.002

Elevation of BFT	2.69	1.17–6.2	0.020

BMI >25	1.63	0.73–3.63	0.235

BMI, body mass index; BFT, biliary function test.

## Data Availability

The data used to support the findings of this study may be released upon application to the institutional review board committee at the Kariya Toyota General Hospital that can be contacted at corresponding author.
